# Green Synthesis of a Carbon Quantum Dots-Based Superhydrophobic Membrane for Efficient Oil/Water Separation

**DOI:** 10.3390/ma16155456

**Published:** 2023-08-03

**Authors:** Rasmiah Saad Almufarij, Mohamed Elshahat Mohamed

**Affiliations:** 1 Department of Chemistry, College of Science, Princess Nourah bint Abdulrahman University, P.O. Box 84428, Riyadh 11671, Saudi Arabia; rsmufrrig@pnu.edu.sa; 2 Chemistry Department, Faculty of Science, Alexandria University, Alexandria 21568, Egypt; 3 Faculty of Advanced Basic Sciences, Alamein International University, Alamein City 51718, Egypt

**Keywords:** carbon quantum dot, eco-friendly, superhydrophobic membrane, oil/water separation, flux rate, chemical stability

## Abstract

The efficient separation of oil and water is a significant challenge worldwide due to the increasing frequency of industrial oily wastewater. Previous work by our group utilizes biological metal–organic framework-based superhydrophobic (S.P) textile fabric for oil/water separation. However, this system is limited due to the low mechanical stability, so there is a need for producing a more robust S.P membrane for oil/water separation. In this study, we report on the synthesis of carbon quantum dots (CQD) from banana leaves via a hydrothermal process and their application in producing a robust S.P coating on textile fabric for oil/water separation. The CQDs were characterized using various techniques including TEM, XRD, absorbance spectroscopy, and the BET method. The TEM images showed that the CQDs were circular in shape with a size of 4.4 nm, while the XRD micrograph indicated that the CQDs were crystalline in nature. The UV–vis graph showed a peak at a wavelength of 278 nm, suggesting strong absorption in the ultraviolet region. The BET-specific surface area of the prepared CQDs is 845 m^2^/g, with a pore volume of 0.33 cm^3^/g, and a mean pore diameter of 1.62 nm. We examined the surface wettability, morphology, composition, oil absorption capacity, oil/water separation performance, flux rate, chemical stability, and mechanical stability of the S.P membrane. Our findings indicate that the developed CQD-based S.P membrane possesses excellent S.P properties, displaying high water contact angles of 163° and low water sliding angles of 1°. The membrane demonstrated superior oil absorption capacity, separation efficiency, and flux rate towards three different oils—petroleum ether, n-hexane, and silicone oil. Petroleum ether has the highest separation efficiency (99.5%), and flux rate (13,500 L m^−2^ h^−1^), while silicone oil has the lowest. However, silicone oil has the highest absorption capacity (218.9 g/g) and petroleum ether has the lowest (194.8 g/g). For the absorption capacity and separation efficiency, a one-way ANOVA test was conducted. The statistical analyses revealed significant differences in absorption capacity and separation efficiency for the three oils, highlighting the efficacy of the superhydrophobic membrane for tailored oil/water separation. Additionally, the S.P membrane exhibited good mechanical (the membrane maintains its superhydrophobicity until an abrasion length of 850 cm) and chemical stability (the membrane maintains its superhydrophobicity in pH range 1–13), withstanding abrasion and immersion in solutions of varying pH values. The CQD-based S.P membrane shows great potential as a promising material for oil/water separation applications, with excellent performance and stability under various environmental conditions.

## 1. Introduction

Modern human society faces a critical and difficult requirement for a sustainable supply of clean water. The discharge and leakage of oily waste materials into several water streams (including seawater, groundwater, and freshwater) have become a significant challenge due to the industrial revolution [1,2]. The production of oily wastewater from industries such as petrochemical finishing, leather, food, and textile has become a major pollutant globally [3,4]. Additionally, crude oil falls throughout marine transportation or offshore oil production have resulted in the release of millions of tons of liquid petroleum hydrocarbon into the coastal areas or ocean, which leads to significant economic losses and poses a direct threat to communities, public health, and environmental systems. Therefore, there is a crucial need to develop materials and technologies for separating oil and water (O-W) [5,6].

Various conventional methods have been utilized to separate mixtures of O-W. These methods include physical techniques such as ultrasonic separation, dissolved air floatation, centrifugation, and gravity separation, as well as chemical techniques such as flocculation separation, neutralization, and coagulation [7,8,9,10]. Electrochemical techniques such as electrocoagulation separation and electro flocculation, mechanical methods such as skimmers and booms, and biological treatment methods such as activated sludge, biological rotating disc process, and bioaugmentation have also been employed [11,12,13,14]. Despite their usefulness, these separation methods have certain drawbacks. For instance, they may occupy a large footprint, require high energy consumption, lead to secondary pollution, and have low separation efficiency [1]. As a result, these methods are not ideal for scenarios such as continuous production processes or large oil spills that generate high volumes of oily wastewater. Therefore, there is a growing interest in exploring new alternative functional materials that can facilitate cost-effective and efficient O-W separation.

Researchers have conducted extensive studies on wettable membranes inspired by nature for O-W separation [15,16]. Recently, there has been a lot of interest in developing advanced and multifunctional materials with biomimetic designs [1]. Membranes with superhydrophobic (S.P) properties, characterized by a water contact angle (WCA) greater than 150 degrees and a water sliding angle (WSA) less than 10 degrees, can repel water droplets while enabling oil to pass through them c. As a result, they are highly suitable for separating oil and water.

S.P materials have demonstrated significant potential in various applications, including corrosion protection of metals and alloys, drag reduction, self-cleaning, anti-icing, and O-W separation [17,18,19,20,21]. Researchers have taken inspiration from natural surfaces such as rose petals, lotus leaves, geckos, and water striders to create S.P surfaces [1]. They achieve this by constructing graded rousgh structures using micro/nanomaterials (e.g., metal oxide nanoparticles, metal nanoparticles, and carbon nanomaterials) and then altering the rough surface with low surface energy substances (such as fatty acids, polydimethylsiloxane, and organosilanes) [22]. Numerous materials, such as a sponge, metal mesh, and fabric, have been investigated for their exceptional super-wetting or super-anti wetting properties in separating O-W mixture [17].

In recent times, different techniques such as plasma etching, chemical vapor deposition, the hydrothermal method, electrodeposition, and atomic layer deposition process have been employed to create S.P membranes [23,24,25]. However, these methods are complicated manufacturing processes or time-consuming, while dip-coating is considered as a simple and scalable method for fabricating S.P membranes [26]. Its scalability and versatility for O-W separation applications are due to its ability to produce functional coatings on nearly any substrate on a large scale.

Carbon quantum dots (CQDs) are nanomaterials with carbon skeleton structures that have functional groups on their surfaces and are often spherical in shape and less than 10 nm in size [27,28]. CQDs were discovered accidentally during the electrocatalytic process of carbon nanotubes in 2004. Since then, they have garnered significant attention as innovative carbon materials with vast application potential. CQDs are considered zero-dimensional carbon materials and are now being utilized to improve health protection, environmental management, energy utilization efficiency, and precision instrumentation technologies. Due to their abundant surface functional groups and nanometer size, CQDs dissolve easily in water and are simple to functionalize. Additionally, CQDs typically consist of carbon (C), oxygen (O), and hydrogen (H) elements. One promising approach for CQD synthesis involves utilizing biomass waste as a raw material source [29]. Biomass waste, which includes agricultural and industrial waste products, is a renewable and sustainable resource that is abundant and cost-effective. By converting these waste products into CQDs, researchers can create value-added products while also reducing waste and promoting environmental sustainability. CQDs have been synthesized by various methods, including ultrasonication, hydrothermal, microwave, electrochemical, and laser ablation [30]. The hydrothermal method is preferred due to its low cost, convenience, and mild reaction condition [31].

Fluorinated chemicals, such as fluorocarbon molecules or fluorosilane, are commonly used to create S.P coatings due to their extremely low surface energy [22]. However, they pose a significant risk to human health and the environment where studies have shown that the utilization of fluorocarbons with long chains has detrimental effects, including bioaccumulation, biomagnification, and persistence [22]. As a result, it remains a challenge to develop environmentally friendly strategies for producing superhydrophobic/superoleophilic fabrics that can effectively separate O-W or absorb oil while overcoming these limitations.

Previous work by our group utilized metal–organic framework-coated textiles for oil/water separation [17]. However, it has low mechanical stability. The objective of this study is to create a robust CQD-based S.P coating on textile fabric (TF) for O-W separation. CQDs are synthesized from biomass waste materials obtained from banana leaves using a hydrothermal process. Stearic acid (S.A) is used as a low surface energy material due to its affordability and environmentally friendly nature. The chemical composition, size, and morphology of the CQDs are analyzed. Additionally, the surface composition, morphology, wettability, oil absorption capacity, O-W separation performance, flux, and mechanical and chemical stability of the prepared superhydrophobic textile fabric (STF) are examined.

## 2. Materials and Methods

### 2.1. Materials

The experiment employed analytical-grade sulfuric acid, sodium hydroxide, n-hexane (n-H.E), petroleum ether (P.E), silicone oil (S.O), bromothymol blue, and stearic acid (S.A), which were purchased from Chematek company (Egypt). The pristine textile fabric (TF) and banana leaves were bought from a nearby marketplace.

### 2.2. The Preparation Method

#### 2.2.1. Synthesis of Carbon Quantum Dot (CQD)

The typical procedure involved washing 2 g of banana leaves with distilled water twice and air-drying them. The dried leaves were then finely powdered using a mortar and pestle. The resulting powder was mixed with 30 mL of distilled water and stirred at room temperature for 15 min. The solution was then transferred into a Teflon autoclave and heated at 200 °C for 3 h. After cooling to room temperature, a carbonaceous solution was obtained and centrifuged at 10,000 rpm for 15 min to remove larger and undissolved particles, and the supernatant solution was collected. The supernatant solution was then passed through a 0.22 μm syringe filter, resulting in a light brown solution that was further diluted with ultrapure water for subsequent use.

#### 2.2.2. Fabrication of Superhydrophobic Textile Fabric (STF)

A circular TF measuring 10.5 mm in diameter was placed in a solution of carbon quantum dots (CQDs) and left to soak for one hour. The TF was then air-dried for 2 h at room temperature and subsequently dried for 2 h at a temperature of 100 °C. Next, the TF was submerged in an ethanolic solution containing 0.01 M S.A for 0.5 h. The resulting superhydrophobic textile fabric, TF@CQD@S.A, was air-dried for 2 h at room temperature and then dried for 2 h at a temperature of 60 °C.

### 2.3. Characterization of the Prepared CQDs

The absorption behavior of the prepared CQD was investigated using a UV–vis spectrophotometer with a scan range of 200 to 400 nm. Before measuring the spectra, the samples were diluted in distilled water. All measurements were conducted at room temperature. The size and shape of CQS were determined using a transmission electron microscope (TEM-1400 Plus Electron Microscope, JEOL, Tokyo, Japan). A Bruker D2 Phaser X-ray diffractometer was utilized to perform an X-ray diffraction examination using Cu K radiation with a monochromatic source. The composition of the prepared CQD was analyzed using Fourier-transform infrared spectroscopy (FTIR) (model: Bruker Tensor 37 FTIR). The Brunauer–Emmett–Teller method (BET-Beckman coulter, SA3100, Krefeld, Germany) was used to investigate the textural properties of the prepared CQD.

### 2.4. Characterization of the Pristine and Modified Membrane

To examine the morphological changes in the TF following S.P coating, a JEOL JSM-200 IT scanning electron microscope (SEM) was utilized. The elemental composition of the TF before and after S.P coating was analyzed using Fourier-transform infrared spectroscopy (FTIR) (model: Bruker Tensor 37 FTIR). We utilized an energy-dispersive X-ray spectrometer (EDX JEM-2100 Japan, JEOL, Tokyo, Japan) to determine the elemental composition of the CQDs, and the TF before and after S.P coating. The water contact angle (WCA) and water sliding angle (WSA) were measured with a Rame-hart WCA instrument (model 190-F2) using 5 µL water droplets, and the reported WCA values are the average of four tests conducted at different locations on the prepared STF surface. The pH of the water droplets was adjusted using sulfuric acid and sodium hydroxide.

To evaluate the mechanical stability of the prepared STF, an abrasion test was performed. The STF was placed on 800-mesh sandpaper and dragged with a weight that exerted a pressure of 5 kPa, and the WCA and WSA were measured every 5 cm. The chemical stability of the STF was assessed by immersing several fabricated samples in solutions with a pH range of 1 to 13 for one hour, and the impact on the WCA and WSA values was evaluated. The reported mechanical and chemical stability data represent the average of three tests and the standard deviation error bar is represented in the graphs.

### 2.5. Absorption Capacity and Separation Performance

When evaluating absorbing materials, absorption capacity is a crucial factor in practice. To determine the adsorption capacity of the STF, a typical absorption test was conducted. Initially, the weight of a clean STF was recorded in the air as M_0_ (g). The STF was then placed in oil or organic solvents until it reached adsorption saturation, and it was reweighed again as M_1_ (g). The adsorption capacity (Q, g/g) of the STF was calculated using Equation (1) [32]:

Q = M_1_/M_0_ (1)

To assess the oil/water separation performance of the STF, a mixture of 20 mL organic solvent and 20 mL water (bromothymol blue was added to the distilled water to provide color) was poured onto a self-built gravity-driven laboratory device. While the TF as a filtration membrane allows the passage of both oil and water, the STF as a filtration membrane allows the passage of oil only and so separation of O-W mixture. Upon introducing oily wastewater into the separation system, the STF facilitated the passage of oil, which accumulated in the lower container, while the water was retained in the upper container. The weight of the oil before (m_0_) and after (m_1_) separation was measured to determine the separation efficiency (η) using Equation (2) [32].

η = (m_1_/m_0_) × 100% (2)

To determine the flux rate of the STF, which refers to the rate at which the oil passes through a membrane per unit area per unit time, Equation (3) was employed [32].

Flux = V/(S × t) (3)

**Figure 1:**
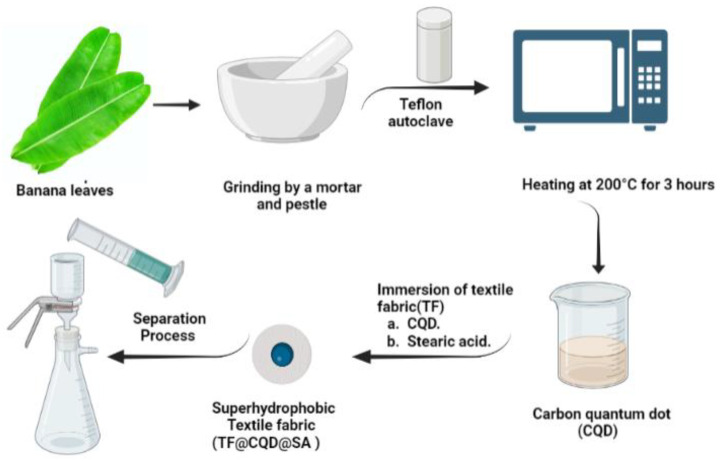
The synthesis of CQD using hydrothermal process and the fabrication of STF and its utilization in O-W separation.

Here, V (L) denotes the volume of oil permeation, S (m^2^) is the contact area, and t (h) represents the recorded oil permeation time. The oil absorption capacity and oil/water separation performance tests were conducted three times and the standard deviation error bar is represented in the graphs. A schematic illustration of the synthesis of CQD and fabrication of STF and its utilization in the separation procedure is presented in Figure 1.

### 2.6. Statistical Analysis

The statistical analysis for the absorption capacity and separation performance tests for the prepared STF toward the three oils was performed using descriptive statistics and a one-way analysis of variance (ANOVA) test. The data obtained for each trial (three trials were performed) of absorption capacity and separation performance for each oil were recorded and analyzed. The control group consisted of pristine textile fabric without any superhydrophobic coating. Descriptive statistics were used to calculate the mean and standard deviation of the absorption capacity and separation performance for each oil. This helped in summarizing the data and understanding the differences in absorption capacity between the superhydrophobic fabric and the control group. To determine if there were significant differences in the absorption capacity and separation performance between the three oils, a one-way ANOVA test was conducted. The null hypothesis (H0) stated that there were no significant differences between the means of the groups, while the alternative hypothesis (HA) hypothesized significant differences between the means. The ANOVA results provided an F-value and a *p*-value. The F-value indicated the variability between the groups compared to the variability within the groups. The *p*-value determined the statistical significance of the observed differences. A significance level (α) of 0.05 was chosen to assess the results. If the *p*-value was less than this significance level, it was considered statistically significant, leading to the rejection of the null hypothesis.

## 3. Results and Discussion

### 3.1. Characterization of the CQD

**Figure 2:**
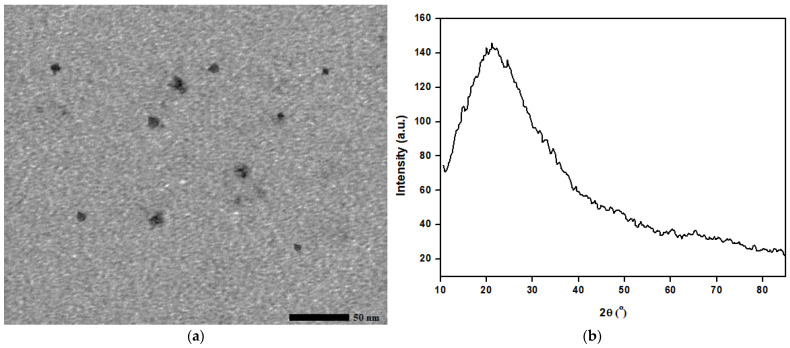

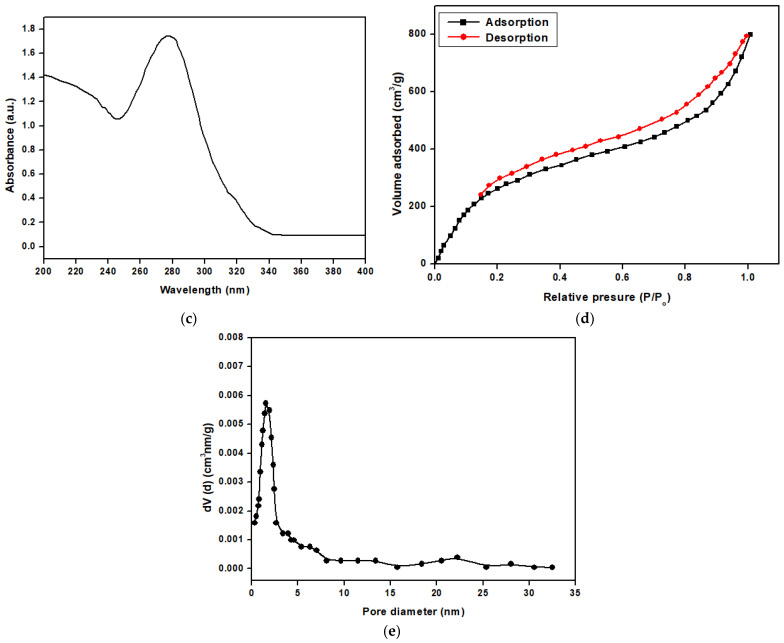
(**a**) TEM micrograph, (**b**) XRD micrograph, (**c**) UV–vis spectrum, (**d**) BET surface area, and (**e**) BET pore size distribution of the prepared CQDs.

The CQDs were characterized using TEM, XRD, absorbance spectroscopy, and the BET method, Figure 2. The TEM technique is used for the visualization of nanoscale materials and can provide information on particle size, and morphology. The TEM images, Figure 2a, showed that the CQDs were circular in shape with a size of 4.4 nm. The XRD technique is used to determine the crystal structure of materials. The XRD micrograph, Figure 2b, shows a broad peak at two theta of 22.1° indicating that the CQDs were crystalline in nature. Absorbance spectroscopy is often used to determine the optical properties of materials by measuring the absorption of light by a material as a function of wavelength. The UV–vis graph, Figure 2c, showed a peak at a wavelength of 278 nm, indicating that the CQDs had strong absorption in the ultraviolet region, likely due to the n-π* transition of the carbon core of the CQDs. Figure 2d shows the BET-specific surface area of the prepared CQDs as demonstrated by the N_2_ adsorption/desorption isotherm. Figure 2e shows the pore size distribution of the prepared CQDs. The BET-specific surface area of the prepared CQDs is 845 m^2^/g, the pore volume is 0.33 cm^3^/g, and the mean pore diameter of 1.62 nm. When CQDs are grafted onto a substrate, a larger BET surface area of the CQDs will result in the creation of more roughness on the substrate surface (the roughness is the parameter for creating an S.P surface). The yield of prepared CQDs is 13.5%.

### 3.2. Characterization of the Membrane

#### 3.2.1. FTIR Results

**Figure 3:**
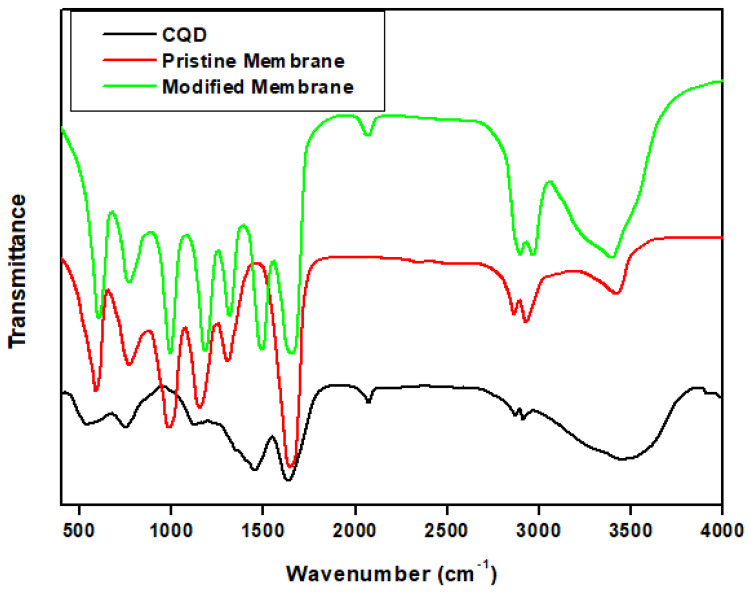
FTIR of the prepared CQDs, TF and STF.

FTIR is a commonly used spectroscopic technique that provides information on the chemical bonds present in a material. The FTIR results for CQDs, TF, and STF are shown in Figure 3. The FTIR results for the CQDs show numerous peaks which correspond to various functional groups that are present in the CQDs. The broad peak at 3463 cm^−1^ is likely due to the presence of O-H stretching vibrations [33]. The peaks at 2929 cm^−1^ and 2861 cm^−1^ correspond to C-H stretching vibrations, while the peak at 2072 cm^−1^ is likely due to C≡C stretching vibrations [34]. The peaks at 1631 cm^−1^ and 1442 cm^−1^ correspond to C=C stretching vibrations, while the peak at 1126 nm is due to C-O stretching vibrations. The peaks at 754 cm^−1^ and 550 cm^−1^ correspond to C-H bending vibrations [35].

The FTIR results for the TF show various peaks which correspond to various functional groups that are present in the TF. The peak at 3414 cm^−1^ is attributed to the N-H stretching vibrations. The peaks at 2930 cm^−1^ and 2862 cm^−1^ are due to the symmetric and asymmetric stretching vibration of CH. The peak at 1643 cm^−1^ is likely due to C=C stretching vibrations. The peaks at 1305 cm^−1^ and 1159 cm^−1^ are due to the C-N stretching vibration. The peak at 989 cm^−1^ is attributed to the bending of C-H. The peak at 764 cm^−1^ is due to the out-of-plane bending of N-H while the peak at 595 cm^−1^ may correspond to C-H out-of-plane bending vibrations [17].

The FTIR results for the STF show similar peaks to that of CQDs and TF with a small shift in peak position indicating that the TF is successfully grafted with CQDs and stearic acid.

#### 3.2.2. EDX Results

**Figure 4:**
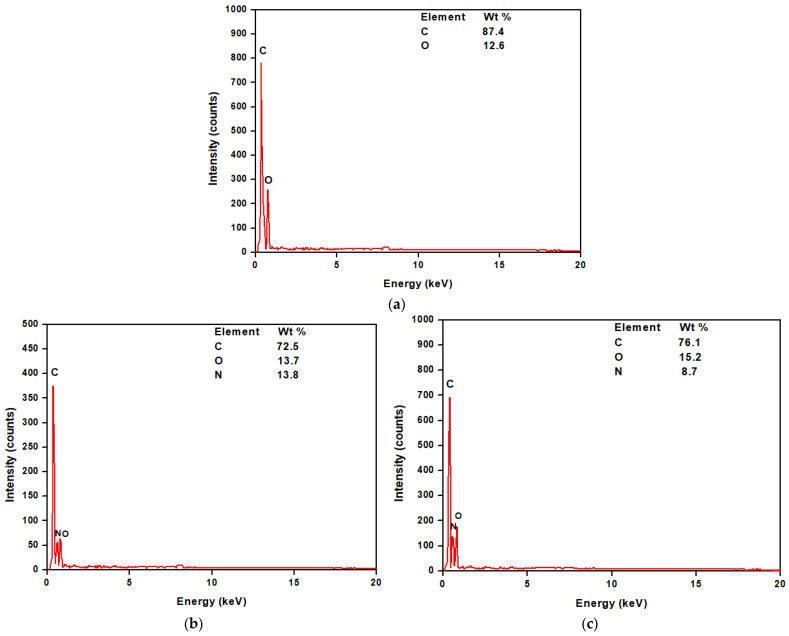
The EDX micrograph for the (**a**) prepared CQDs, (**b**) TF, and (**c**) STF.

The EDX technique is utilized to investigate the composition of the CQD, TF, and STF, Figure 4. The results showed that CQD has peaks of C and O, this result confirms that obtained from FTIR. The TF had peaks of C, N, and O. However, the EDX for STF displayed peaks for C, N, and O with higher intensity and a higher weight (wt) % of C and O atoms indicating that the CQDs and stearic acid were successfully incorporated into the membrane. The EDX results confirm the successful modification of the membrane with the CQD and stearic acid materials.

#### 3.2.3. SEM and the Wettability Results

**Figure 5:**
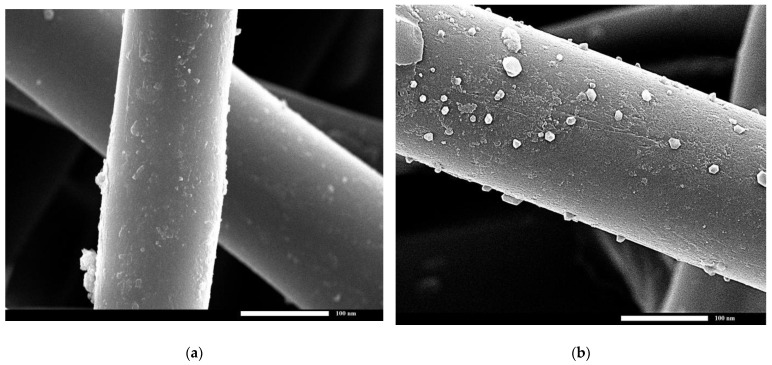
The SEM micrograph for (**a**) TF and (**b**) STF.

The morphology of both the TF and STF membrane was examined using SEM, as depicted in Figure 5. The SEM results revealed that the TF had a smooth structure, while the STF exhibited rough structures. This can be attributed to the presence of CQDs, which are small in size and can create a rough surface. The presence of a rough surface is a crucial characteristic of an S.P coating.

To assess the wetting behavior of the TF and STF, the WCA and WSA were measured. The TF exhibited hydrophilic properties, with a WCA value equal 0 degrees. This caused water droplets to adhere to the surface without sliding off, even when the fabric was tilted. In contrast, the STF demonstrated remarkable S.P properties, with a WCA value of 163° ± 0.7° and a WSA value of 1° ± 0.1°.

### 3.3. Absorption Capacity Measurements

**Figure 6:**
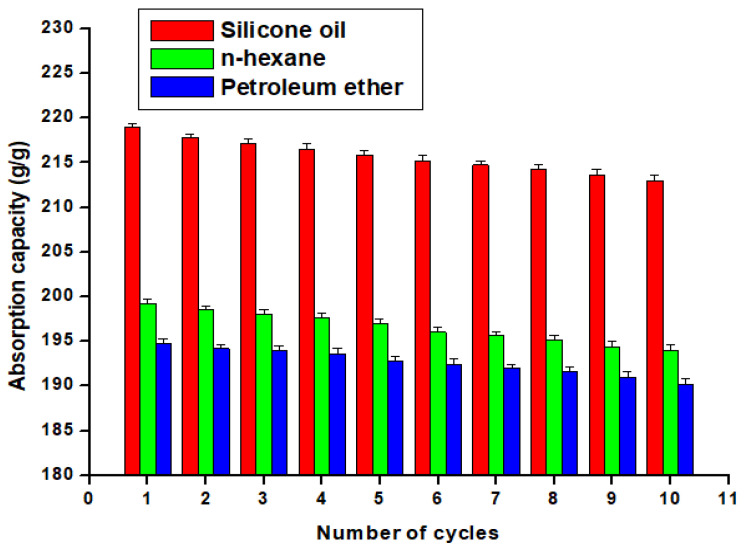
Variation of the absorption capacity with the number of cycles of the prepared STF.

Figure 6 illustrates that the STF absorption capacity for S.O was higher than that of n-H.E and P.E. This indicates that the STF has a greater affinity for S.O than the other two oils. Studies have shown that an increase in oil viscosity and density can enhance absorption capacity [36,37,38,39,40]. The STF, with its low density, high porosity, S.P properties, and capillary force, can rapidly absorb organic solvents [32].

To evaluate the stability of the STF, its absorption capacity towards the three oils was measured ten times. After each cycle, the oil absorption capacity was determined, and the STF was compressed to ensure complete desorption of the organic solvent. The absorption capacity of the STF towards the three oils remained constant after ten cycles of absorption and desorption. This suggests that the STF is stable and can be reused multiple times without losing its oil absorption capacity. The prepared STF has superior absorption capacities compared to previously known absorbents [41,42].

Table 1 shows the statistical analysis on the absorption capacity data for the three oils. From the descriptive statistics, we can see that the STF has the highest mean absorption capacities toward the S.O while has the lowest mean absorption capacities toward P.E. By conducting a one-way ANOVA test, we can determine if there are significant differences in absorption capacity between the three oils. The obtained *p*-value is less than the significance level (α = 0.05), indicating that there is strong evidence to reject the null hypothesis. This means that there are significant differences in absorption capacity between the three oils.

### 3.4. O-W Separation Efficiency

**Figure 7:**
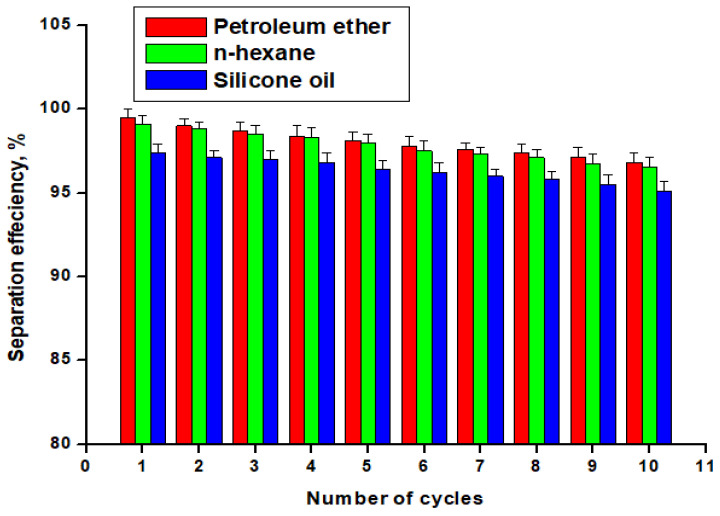
The separation efficiency of the STF with the separation cycles number for a mixture of O-W.

Figure 7 depicts the O-W separation efficiency of the developed STF for ten cycles of use with three different oils: P.E, n-H.E, and S.O. The results indicate that the separation efficiency of P.E is the highest among the three oils, with a separation efficiency of 99.5%, followed by n-H.E with a separation efficiency of 99.1%, and S.O with a separation efficiency of 97.4%. The higher separation efficiency for P.E could be attributed to its lower surface tension and viscosity compared to n-H.E and S.O, making it easier to separate from water.

The separation efficiency of the STF decreased gradually with the number of cycles, and the separation efficiency reached a minimum value of approximately 95% after ten separation cycles. This decrease in separation efficiency could be due to the accumulation of oil on the surface of the STF, which can clog the pores and reduce its ability to repel water. Additionally, repeated cycles of absorption and desorption can cause wear and tear on the coating, leading to a decline in its performance over time. The O-W separation efficiency of the STF is higher than that of previously reported absorbents [43,44].

Table 2 shows the statistical analysis on the separation efficiency of the STF for the three oils. From the descriptive statistics, we can observe that the P.E has the highest separation efficiency while S.O has the lowest separation efficiency. By conducting a one-way ANOVA test, we can determine if there are significant differences in absorption capacity between the three oils. The obtained *p*-value is less than the significance level (α = 0.05), indicating strong evidence to reject the null hypothesis. Thus, it can be concluded that there are significant differences in separation performance between the three oils.

### 3.5. Mechanical Stability

**Figure 8:**
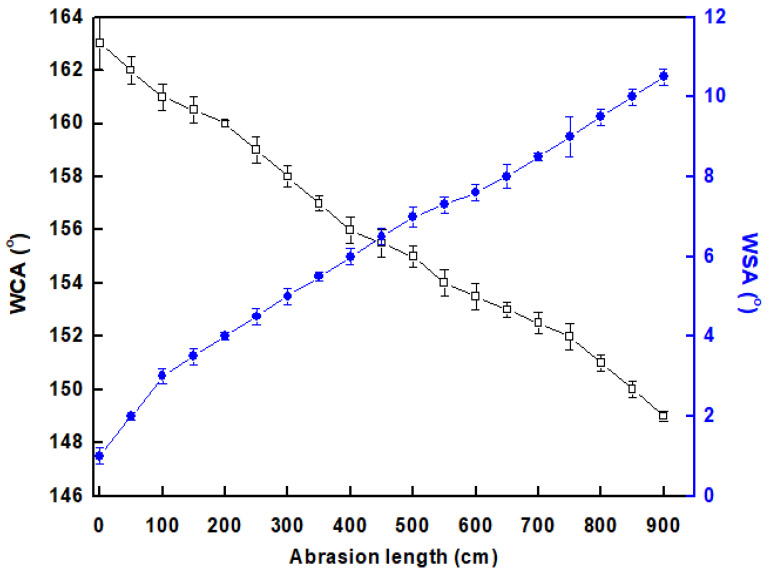
Change in the water contact angle (WCAs) and water sliding angle (WSAs) with the length of abrasion of STF on 800-mesh sandpaper.

The mechanical stability of the developed STF was examined by evaluating the impact of abrasion length on the WCA and WSA on the STF, as shown in Figure 8. The results reveal that as the abrasion length increases, the WCA decreases, and the WSA increases, indicating a reduction in the membrane’s superhydrophobicity. However, the membrane maintains its superhydrophobicity until an abrasion length of 850 cm, where the WCA remains higher than 150 degrees, and the WSA remains lower than 10 degrees. This indicates that the STF has good mechanical stability and can withstand a certain level of wear and tear while retaining its S.P properties. The STF exhibits enhanced mechanical stability compared to many previously reported values [42,45].

### 3.6. Chemical Stability and Flux Rate

**Figure 9:**
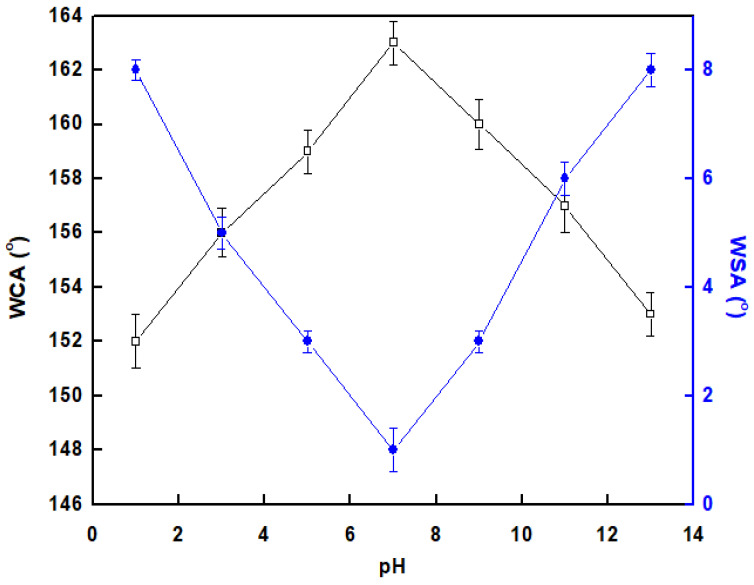
The variation of the water contact angle (WCA) and water sliding angle (WSA) after one-hour immersion of the prepared STF in solutions of varying pH.

The chemical stability of the developed STF under different pH conditions was studied, Figure 9. The results suggest that the membrane is generally chemically stable and can maintain its S.P properties under different pH conditions. At pH range 1–13 the prepared STF exhibits S.P property where it always has a WCA greater than 150° and a WSA less than 10°. These results suggest that the developed STF membrane has good chemical stability under neutral, basic, and acidic conditions.

The flux rate of the developed STF towards three different oils, namely n-H.E, P.E, and S.O, was examined. The results indicate that the flux rate of the STF towards P.E is the highest among the three oils, with a value of 13,500 L m^−2^ h^−1^, followed by n-H.E with a value of 13,100 L m^−2^ h^−1^, and S.O with a value of 11,900 L m^−2^ h^−1^. This suggests that the membrane has higher permeability towards P.E due to its lower viscosity and surface tension and viscosity compared to the other oils.

The impact of repeated separation cycles on the flux rate of the membrane was investigated. The results reveal that after ten separation cycles, the flux values for the three oils have slightly decreased, with the flux rate for P.E decreasing to 12,850 L m^−2^ h^−1^, n-H.E to 12,600 L m^−2^ h^−1^, and S.O to 11,100 L m^−2^ h^−1^. This decrease in flux rate could be attributed to the accumulation of oil on the surfasce of the coating, which can clog the pores and reduce their permeability. The flux rates of the STF are superior to those of many previously reported absorbents [46].

## 4. Conclusions

The separation of oil and water is an important challenge due to industrial oily wastewater. Prior work by our group used metal–organic framework (MOF)-coated textiles fabric for oil/water separation. However, the mechanical stability was low. In this work, a robust CQDs-based S.P coating was successfully fabricated on textile fabric for O-W separation. The CQDs were prepared using banana leaves as a raw material by the hydrothermal method.

The prepared CQDs were characterized using multiple techniques, including transmission electron microscopy (TEM), X-ray diffraction (XRD), absorbance spectroscopy, and the BET method. TEM analysis revealed that the CQDs exhibited a circular shape with an average size of 4.4 nm. XRD analysis indicated that the CQDs possessed a crystalline structure. The UV–vis spectrum exhibited a pronounced peak at 278 nm, suggesting strong absorption in the ultraviolet region. Furthermore, the BET analysis determined that the prepared CQDs had a specific surface area of 845 m^2^/g, a pore volume of 0.33 cm^3^/g, and a mean pore diameter of 1.62 nm. The surface composition, morphology, wettability, O-W separation performance, oil absorption capacity, flux rate, mechanical stability, and chemical stability of the membrane were investigated. The results indicate that the developed STF membrane exhibits excellent S.P properties, with a high WCA of 163 degrees and a low WSA of 1 degree. The membrane also shows good oil absorption capacity, separation efficiency, and flux rate towards three different oils, namely n-H.E, P.E, and S.O. The P.E has the highest separation efficiency and flux rate, while the S.O has the lowest. On the other hand, the S.O has the highest absorption capacity, and the P.E has the lowest. The statistical analyses were performed using a one-way ANOVA test on both the absorption capacity and separation efficiency data. The obtained *p*-values were significantly lower than the predetermined significance level, indicating robust evidence supporting the presence of significant differences among the three oils. These findings highlight the effectiveness of the superhydrophobic membrane for oil/water separation, with varying performance depending on the type of oil. This information is crucial in the development of efficient and tailored separation materials for specific oil/water separation applications. The membrane also demonstrates good mechanical and chemical stability, with the ability to withstand abrasion and immersion in solutions of different pH values for varying immersion times.

## Figures and Tables

**Table 1:** The statistical analysis on the absorption capacity of the STF for the three oils.

Oil Name	Mean (g/g)	Standard Deviation	F-Value	*p*-Value
S.O	218.9	1.24	233.9	<0.001
n-H.E	199.2	1.07		
P.E	194.8	0.69		

**Table 2:** The statistical analysis on the separation efficiency of the STF for the three oils.

Oil Name	Mean (g/g)	Standard Deviation	F-Value	*p*-Value
S.O	97.4	0.70	5373	<0.001
n-H.E	99.1	0.25		
P.E	99.5	0.40		

## Data Availability

All data in this study will be available upon request.
